# Exploring Upward and Downward Provider Biases in Family Planning: The Case of Parity

**DOI:** 10.9745/GHSP-D-22-00470

**Published:** 2023-06-21

**Authors:** Brooke W. Bullington, Nathalie Sawadogo, Katherine Tumlinson, Ana Langer, Abdramane Soura, Pascal Zabre, Ali Sié, Leigh Senderowicz

**Affiliations:** a Department of Epidemiology, Gillings School of Global Public Health, University of North Carolina at Chapel Hill, Chapel Hill, NC, USA.; b Carolina Population Center, University of North Carolina at Chapel Hill, Chapel Hill, NC, USA.; c Institut Supérieur des Sciences de la Population, Université Joseph Ki-ZERBO, Ouagadougo, Burkina Faso.; d Department of Maternal Child Health, Gillings School of Global Public Health, University of North Carolina at Chapel Hill, Chapel Hill, NC, USA.; e Department of Global Health and Population, Harvard T.H. Chan School of Public Health, Harvard University, Boston, MA, USA.; f Centre de Recherche en Santé de Nouna, Nouna, Burkina Faso.; g Department of Gender and Women’s Studies, University of Wisconsin-Madison, Madison, WI, USA.; h Department of Obstetrics and Gynecology, University of Wisconsin-Madison, Madison, WI, USA.

## Abstract

**Introduction:**

Provider bias has become an important topic of family planning research over the past several decades. Much existing research on provider bias has focused on the ways providers restrict access to contraception. Here, we propose a distinction between the classical “downward” provider bias that discourages contraceptive use and a new conception of “upward” provider bias that occurs when providers pressure or encourage clients to adopt contraception.

**Methods:**

Using cross-sectional data from reproductive-aged women in Burkina Faso, we describe lifetime prevalence of experiencing provider encouragement to use contraception due to provider perceptions of high parity (a type of upward provider bias) and provider discouragement from using contraception due to provider perceptions of low parity (a type of downward provider bias). We also examine associations between sociodemographic characteristics and experiences of provider encouragement to use contraception due to perceptions of high parity.

**Results:**

Sixteen percent of participants reported that a provider had encouraged them to use contraception due to provider perceptions of high parity, and 1% of participants reported that a provider had discouraged them from using contraception because of provider perceptions of low parity. Being married, being from the rural site, having higher parity, and having attended the 45th-day postpartum check-up were associated with increased odds of being encouraged to use contraception due to provider perceptions of high parity.

**Conclusion:**

We find that experiences of upward provider bias linked to provider perceptions of high parity were considerably more common in this setting than downward provider bias linked to perceptions of low parity. Research into the mechanisms through which upward provider bias operates and how it may be mitigated is imperative to promote contraceptive autonomy.

## Abstract (precis)

The authors conceptualize a distinction between “upward” provider bias that occurs when providers pressure or encourage clients to adopt contraception and “downward” provider bias in family planning that discourages contraceptive use.

## Teaser

**Key Findings:**

- “Upward” and “downward” provider biases are distinct manifestations of provider bias when providers pressure or encourage clients to adopt contraception (upward bias) or when providers hinder family planning access (downward bias).
- Seeking to expand the understanding of provider bias, we analyzed cross-sectional survey data of reproductive-aged women in Burkina Faso and found that 16% of women reported that providers had encouraged them to use contraception because of high parity (upward provider bias) and 1% of women reported providers had discouraged them from using contraception because of low parity (downward provider bias).
- Age, marital status, number of children, rurality, and attendance at a 45th-day postpartum visit were all associated with provider encouragement to use contraception due to high parity.

**Key Implication:**

- Further research is needed to understand the mechanisms through which upward provider bias operates and develop interventions to address provider bias to ensure contraceptive autonomy for all people.

## INTRODUCTION

### Provider Bias in Family Planning Research

Over the past 4 decades, provider bias has emerged as a central topic in family planning (FP) research. In 1985, Schuler et al. were among the first researchers to describe and empirically measure bias in patient-provider interactions in FP clinics. Using mystery clients in Nepal, the team reported that “lower class” patients were less likely to receive accurate information and high-quality treatment compared to “middle class” clients, indicating bias from providers based on clients’ socioeconomic position.^1^ Bruce incorporated this “bias of the provider” in her 1990 landmark paper on quality of care, with Schuler et al.’s findings part of the foundation for the interpersonal relations pillar of Bruce’s quality framework.^2^ In 1992, Shelton et al. published an article on medical barriers to FP access, delving further into provider bias. Shelton et al. argued that provider bias is deeply intertwined with economic and political interests and can serve as a mechanism that translates ideological opposition to contraception into restricted access.^3^

In the years since Shelton formalized the concept of provider bias, the topic has gained increasing prominence in FP research. Yet, the construct’s definition remained imprecise until an influential 2019 review by Solo and Festin defined provider bias as “attitudes and subsequent behaviors that unnecessarily restrict client access and choice, often related to either client and/or contraceptive method characteristics.”^4^ In addition to these recent efforts to more clearly conceptualize and define provider bias, researchers have also sought to measure provider bias empirically in countries including the United States,^5^ India,^6^ Nigeria,^7^ Kenya,^8^^,^^9^ Senegal,^10^ and Burkina Faso.^11^ These studies use a range of data collection tools and study designs, including in-depth provider interviews,^5^ surveys, focus group discussions, mystery clients,^1^^,^^9^ patient exit interviews, and discrete choice experiments^11^ to mitigate social desirability bias and attempt to accurately capture patient and provider perspectives on clinical interactions.

The literature has shed considerable light on the scope and magnitude of provider bias, particularly the ways in which provider attitudes can restrict access to desired FP services. For example, multiple studies in Kenya have documented how providers impose restrictions on certain contraceptive methods by requiring a minimum age for provision, refusing to offer methods to nulliparous women, and requiring HIV or pregnancy testing before providing the methods.^8^^,^^9^ A discrete choice experiment in Burkina Faso, Pakistan, and Tanzania reported that marital status and parity may drive provider decisions to counsel and offer contraception more so than age does, although younger women are often most impacted by biased interactions.^11^ A study in urban Nigeria found that levels of provider bias varied depending on which contraceptive method was sought and that in-service training did not substantially reduce biases in provider-patient interactions.^7^ A qualitative study in the United States showed that providers employ a variety of strategies to mitigate potential bias, yet these strategies can exacerbate, rather than reduce, bias in interactions.^11^ With this growing body of methodologically and geographically diverse literature, scholars now better understand the ways sociodemographic characteristics of clients—including age, marital status, and parity—impact provider behavior and reveal bias due to beliefs about who should or should not be contracepting.^4^^,^^6^^,^^7^^,^^9^^,^^10^^,^^12^

### Expanding the Definition of Provider Bias: A Conceptual Framework

Within these studies, the dominant conception of provider bias has remained focused on biases that result in restricted access to contraception. Nearly all studies empirically documenting provider bias measure the ways in which providers prevent women from accessing contraceptive methods due to factors such as young age, low parity, and/or lack of marital union. However, there has been considerably less research into the types of provider bias that lead to unwanted contraceptive use.^5^

The dominant conception of provider bias has focused on biases that result in restricted access to contraception, but there has been less research on provider bias that leads to unwanted contraceptive use.

Drawing from previous work on the construct of contraceptive coercion,^13^ we propose here a distinction between 2 broad categories of provider bias: (1) “downward” provider bias that occurs when providers hinder FP access and choice through withholding or limiting services (in line with the conventional conception of provider bias); and (2) “upward” bias that occurs when providers pressure or encourage clients to use contraception (Box). Both forms of provider bias can occur through selective framing, prodding, judgment, or other means. Similar to contraceptive coercion,^13^ both upward and downward provider biases can fall along a spectrum, ranging from subtle forms of bias in which a provider passes judgment on a patient’s sociodemographic characteristics or contraceptive desires to more overt actions of pressure or force. For example, more subtle downward provider bias might include a provider questioning a client’s method choice because, for example, the client is unmarried but still offering the client their desired method when the client insists that this method is right for them. More overt downward provider bias might include a provider refusing to provide this unmarried client with their desired method altogether.

BOX Definitions of Upward and Downward Provider Biases

**Upward Provider Bias:** Bias that occurs when providers pressure or encourage family planning clients to use contraception (e.g., provider encourages someone to use family planning because they have “too many” children).

**Downward Provider Bias:** Bias that occurs when providers hinder family planning access and choice by withholding or limiting services (e.g., provider discourages someone from using family planning because they have do not have “enough” children).

While downward provider bias has been well explored in FP research, upward provider bias remains an underexamined and potentially important threat to reproductive health and contraceptive autonomy.^14^ Several qualitative studies suggest that FP providers use upward provider bias, through directive and inaccurate contraceptive counseling, to encourage clients to adopt a modern contraceptive method generally and long-acting reversible contraception in particular.^13^^,^^15^^,^^16^ Studies also show that providers may hold biases against long-acting reversible contraception removal, preventing women from making autonomous decisions about contraceptive discontinuation.^13^^,^^17^^,^^18^ However, few studies have attempted to quantify upward provider bias. We posit that upward provider bias may be prevalent in FP programs, especially in the counseling of multiparous women during the postpartum period and for those residing in contexts that have adopted target-based approaches to increasing contraceptive uptake.^19^^,^^20^

### Upward and Downward Provider Biases Due To Parity: A Case Study

Downward provider bias based on providers’ perceptions of a patient’s low parity has been well documented. Nulliparous women or those with few children may be denied a desired contraceptive method because of a provider’s belief that they should have more children before they begin to restrict their fertility or because of a provider’s beliefs about the characteristics or side effects of the client’s preferred method.^8^^,^^9^^,^^21^ In contrast to the scores of studies exploring this dynamic, the ways that multiparity or grand-multiparity may lead to bias in the provision of FP services have scarcely been explored. A qualitative exploration of contraceptive coercion in an anonymized country highlighted an example of this type of upward provider bias, in which after giving birth to her tenth child, a woman was told by health workers that she needed to get the implant “by force” due to her parity.^13^ Although there has been evidence to suggest that upward provider bias is a factor in global FP programs, to our knowledge, there are no studies that attempt to quantify its scope or describe when and why it occurs.

This article is motivated by focus group discussions and in-depth interviews with reproductive-aged women in Burkina Faso who described experiences of upward provider bias based on providers’ perceptions and judgments of their parity. We sought to expand the understanding of provider bias by identifying and measuring a form of more subtle upward provider bias that participants in qualitative interviews frequently described: provider encouragement to use contraception due to perceptions of high parity. Using cross-sectional survey data from reproductive-aged women at 2 sites in Burkina Faso, in this article, we describe lifetime prevalence of experiencing provider encouragement to use contraception because a provider perceived a participant to have “too many” children. We also examine the inverse: a form of downward provider bias focused on provider discouragement away from FP because a provider perceived a participant to not have “enough” children. We explore experiences of contraceptive counseling during postpartum care to further understand when and where upward provider bias occurs. Finally, we examined sociodemographic characteristics associated with experiencing provider encouragement to use contraception based on a provider’s perceptions of parity.

We describe lifetime prevalence of experiencing provider encouragement to use contraception because a provider perceived a woman to have “too many” children and provider discouragement away from FP because a provider perceived a woman to not have “enough” children.

## METHODS

The data for this analysis come from the Contraceptive Autonomy Study, a sequential mixed-methods study that was conducted at 2 sites in Burkina Faso from 2017 to 2018. The first phase of the study, which was qualitative and included 49 in-depth interviews and 17 focus group discussions, was used to develop and refine a novel survey instrument focused on measuring contraceptive autonomy, which was implemented cross-sectionally as the second phase of the study. An initial draft of survey items was shared for feedback with an interdisciplinary range of reproductive health experts from both the Global South (Burkina Faso and Argentina) and the Global North (the United States and Switzerland). An updated version was then translated into French, Mooré, and Dioula for cognitive interviewing, during which a draft version of the survey was shared with 15 reproductive-aged women for verbal feedback. The goal of the cognitive interviews was to determine whether translated survey questions were interpreted the way they were intended.^22^ Cognitive interviews focused on issues of comprehension, recall, judgment, and response.^23^ After further amending the survey tool, it was pilot-tested and updated throughout interviewer training. Standardization of survey questions was central to interviewer training because formal, standardized written translation of the survey into 3 languages was impractical. Data collectors and researchers discussed optimal oral translation of key concepts and questions in-depth. Four days of training were spent on role-playing and practicing interviews, with a specific focus on standardization of language before data collection began. Finally, each data collector conducted 3 pilot surveys that were reviewed individually for feedback and adjustments before data collection. We have described further details about survey design and implementation elsewhere.^24^ The final survey comprised a mix of conventional questions about contraceptive method use adapted from the Demographic and Health Surveys and novel survey questions that sought to measure informed, full, and free contraceptive choice, as well as barriers to autonomous contraceptive decision-making.

### Sampling and Data Collection

The data used in this analysis come from the quantitative phase of this study: a cross-sectional, population-based survey based within the Nouna and Ouagadougou Health and Demographic Surveillance Systems (HDSS). The rural Nouna HDSS is made up of the small administrative town of Nouna and 58 of its surrounding villages, and the urban Ouagadougou HDSS comprises 5 neighborhoods in the northern section of the Burkinabè capital. Full profiles of these HDSSs—including information on their periodicity, demographic makeup, and methods—are available elsewhere.^25^^,^^26^

To be eligible for the survey, women had to live within the catchment area of the Nouna or Ouagadougou HDSS, be of reproductive age (aged 15–49 years, inclusive), and provide informed consent in French, Dioula, or Mooré.^27^ In brief, random samples of reproductive-aged women were drawn in each study site using the Nouna and Ouagadougou HDSS as sampling frames. In Nouna, an initial sample of 2,700 women and 800 potential replacements was drawn, with a response rate of 96.7%. In Ouagadougou, an initial sample of 1,300 women and 700 potential replacements was drawn. Due to a sampling error, the initial sampling frame included 811 women who were “visitors” rather than “residents” in the catchment area and thus ineligible for study inclusion. Thus, a new sample of 500 additional women was drawn. Total response rate in Ouagadougou across both sample drawings was 80.4%. Inverse probability of sampling weights were created and used throughout this analysis to account for changes in the sampling approach.

In total, survey data were collected from 3,929 women between April and July 2018. All adult participants provided written informed consent. For minors, parents provided informed consent with the assent from the minor.

Trained interviewers visited women at their homes and administered the survey orally in French, Mooré, or Dioula, with data recorded on Android-based tablets. Additional detailed information on the sampling strategy of the Contraceptive Autonomy Study can be found elsewhere.^24^

### Analytic Approach

We described sociodemographic characteristics of our sample overall and stratified by site, as well as the characteristics of only women with children. We presented mode of transport as a proxy for household wealth, which is often done in the Burkinabè context.^27^ We examined attendance at the 45th-day postpartum visit. In Burkina Faso, the 45th-day postpartum visit (sometimes called the 40th- or 42nd-day visit) is a check-up that occurs roughly 6 weeks after birth, during which providers are supposed to discuss postpartum FP with clients. Though this visit is recommended for all who deliver, attendance is variable.

Next, we examined the lifetime prevalence of experiences with provider encouragement or discouragement to use or not use contraception due to provider perceptions of parity. We examined the lifetime prevalence of 1 form of downward provider bias—discouragement from using contraception due to provider perceptions of low parity—among all reproductive-aged women using the binary (yes/no) response to the question, “Has a provider ever discouraged you from using a method because they said you don’t have a child or enough children?” The primary outcome of interest was 1 form of upward FP provider bias—encouragement to use contraception due to provider perceptions of high parity. For this outcome, we restricted our sample to women who reported having at least 1 child. Provider encouragement to use contraception due to their perception of high parity was measured via the self-reported, binary (yes/no) response to the question, “Has a provider ever encouraged you to use a method because they said you have too many children?” We did not ask participants to describe how many children they thought providers perceived to be “not enough” or “too many.”

These questions were developed based on in-depth interviews and focus group discussions; therefore, wording of these questions was designed to mirror the ways that women spoke about provider bias they have experienced in their own lives. These measures of upward and downward provider biases were therefore quantified using women’s self-reported experiences with encouragement or discouragement to use contraception based on their parity. Rather than asking women to describe specific experiences with provider bias, our person-centered measurement approach trusted respondents to serve as experts on their own lives and health. Provider encouragement to use contraception during contraceptive counseling may not in and of itself constitute evidence of inappropriately directive counseling. However, our survey items asked specifically about encouragement and discouragement driven by provider perceptions of parity rather than a woman’s own contraceptive desires. This is in line with Solo and Festin’s definition of provider bias,^4^ leading us to suggest provider bias was at play. Women’s perceptions of their interactions with providers offered valuable insights into their experiences.

Women’s perceptions of their interactions with providers offer valuable insights into their experiences.

Informed by our qualitative findings, we also asked those who attended a 45th-day postpartum check-up whether they were asked first if they wanted to use contraception or just told to choose a method. This was determined by the question, “At the 45th-day visit, were you told to just choose a method, or were you asked if you wanted to use a contraceptive method first?” Response options included: told to choose a method, asked to choose a contraceptive method first, asked for a contraceptive method before the provider could mention, and other.

Finally, we used logistic regression to estimate odds ratios to examine sociodemographic factors associated with provider pressure to use contraception due to high parity among women with children. We examined several factors, including age (categorical: 15–24, 25–34, 35–49 years); education level (categorical; none, some primary school, at least some secondary school); marital status (binary; yes/no); primary mode of transport (as a proxy for material wealth; categorical; foot, bicycle, motorcycle, car, other); number of children (categorical: 1–2, 3–4, 5+); site (binary; Nouna, Ouagadougou); and whether an individual had a 45th-day postpartum visit (binary; yes/no). We examined multicollinearity between explanatory variables using variance inflation factor. Because only 41 participants reported that a provider ever discouraged them from using a method because they did not have “enough” children, we did not examine factors associated with this outcome.

### Ethical Approval

This research was reviewed and approved by the Institutional Review Board of the Office of Human Research Administration at the Harvard T. H. Chan School of Public Health in Boston, MA, USA (#IRB17-0511); Le Comité d’Ethique pour la Recherche en Santé du Ministère de la Santé du Burkina Faso in Ouagadougou, Burkina Faso (#2017-5-067); and Le Comité Local d’Ethique du Centre de Recherche en Santé de Nouna, in Nouna, Burkina Faso (#2017-01).

## RESULTS

A total of 3,929 reproductive-aged women were included in our final sample. Demographic characteristics of these women overall and stratified by site are shown in Table 1, along with characteristics of the sample of 2,915 women with children. At both sites, most participants were married (66% in Ouagadougou and 70% in Nouna). Bicycle was the most commonly reported mode of transport in Nouna (70%), whereas motorcycle was most frequently reported in Ouagadougou (69%), indicating higher material wealth among participants from the urban site. Among women with children, many had 3 to 4 children (51% in Ouagadougou and 38% in Nouna), though a far higher percentage of women in Nouna had 5 or more children than women in Ouagadougou (23% vs. 8%). Among those with children, more than half of women in Ouagadougou (63%) and Nouna (54%) reported having previously attended a 45th-day postpartum visit.

**TABLE 1.** Demographic Characteristics of Sample of Reproductive-Aged Women and Reproductive-Aged Women With Children in Nouna and Ouagadougou, Burkina Faso

	All Reproductive-Aged Women			Only Reproductive-Aged Women With Children		
	Ouagadougou, % (n=1,275)	Nouna, % (n=2,654)	Overall, % (n=3,929)	Ouagadougou, % (n=861)	Nouna, % (n=2,054)	Overall, % (n=2,915)
Age, years						
15–24	34	43	40	10	29	23
25–34	33	31	32	42	38	39
35–49	33	27	28	47	33	37
Married	66	70	69	93	86	88
Education						
None	37	56	50	52	66	62
Some primary school	23	24	24	21	25	24
At least some secondary school	36	19	25	22	9	13
Missing	4	0	1	4	0	1
Primary mode of transport						
Foot	3	21	16	4	20	15
Bicycle	14	70	52	14	70	53
Motorcycle	69	8	28	69	9	27
Car	10	0	3	8	0	2
Other	4	1	2	5	1	2
Number of children						
0	35	25	29	0	0	0
1–2	27	29	28	41	38	39
3–4	33	29	30	51	38	42
5+	5	18	13	8	23	19
Contraception use						
Never used	36	60	52	22	53	44
Previously used	30	11	17	37	12	19
Currently using	33	30	31	41	35	37
Attended a 45th day postpartum checkup	43	42	42	63	54	57

Descriptive statistics about lifetime prevalence of provider encouragement or discouragement to use or not use contraception due to parity are shown in Table 2. When asked about experiences with FP providers, 16% (n=460) of women with children said a provider had encouraged them to use a contraceptive method because they had “too many” children. This proportion was considerably higher among women from the rural area of Nouna (19%) compared to those from the capital city (9%). Of the 460 total women who reported experiencing this form of upward provider bias, 42% reported that this occurred within the last year, 25% reported that it occurred within 3 years, and 34% reported that it occurred more than 3 years ago. Approximately 1% (n=41) of all women (not just those with children) in Nouna and Ouagadougou reported that a provider had discouraged them from using a contraceptive method because they did not have “enough” children. Of these 41 women, 29% reported that this occurred within the last year, 11% reported this occurred within 3 years, and 60% reported that it occurred more than 3 years ago.

**TABLE 2.** Reported Lifetime Experiences of Provider Encouragement and Discouragement to Use or Not Use Contraception From Reproductive-Aged Women in Nouna and Ouagadougou, Burkina Faso

	Ouagadougou	Nouna	Overall
Among all reproductive-aged women	n=1,275	n=2,654	n=3,929
Provider discouraged against using contraceptive method because said you did not have “enough” children	1%	1%	1%
Among all reproductive-aged women with children	n=861	n=2,054	n=2,915
Provider encouraged to use contraceptive method because said you had “too many” children	9%	19%	16%
Among reproductive-aged mothers who attended a 45th day postpartum checkup	n=545	n=1,118	n=1,663
At the 45th day visit			
Was told to just choose a method	34%	45%	41%
Was asked if you wanted to use contraception first	61%	31%	41%
Asked for contraception before the provider mentioned it	4%	15%	12%
Other	1%	10%	7%

Details about how providers discussed FP with participants at the 45th-day postpartum visit are also shown in Table 2. In Ouagadougou, most women (61%) were first asked if they wanted to use a contraceptive method, though a considerable percentage (34%) reported that they were just told to choose a method. Four percent of women noted that they asked about a method before the provider brought it up. In Nouna, just less than half (45%) of women were told to just choose a method, and less than one-third (31%) were first asked if they wanted to use a contraceptive method. Fifteen percent of women in Nouna asked for a contraceptive method before the provider mentioned it.

Table 3 shows the results of the logistic regression, describing associations between demographic characteristics and provider encouragement to use contraception because a woman has “too many” children. Odds of experiencing provider encouragement to use contraception due to providers’ perception of high parity were higher among married women compared to unmarried women (adjusted odds ratio [aOR]: 1.56; 95% confidence interval [CI]: 1.08, 2.26). Compared to women aged 15–24 years, women aged 25–34 years (aOR: 1.51; 95% CI: 1.11, 2.06) and 35–49 years (aOR: 1.47; 95% CI: 1.05, 2.07) had higher odds of being encouraged to use contraception due to providers’ perception of high parity. Similarly, the odds of upward provider bias were greater for those from Nouna compared to women from Ouagadougou (aOR: 1.90; 95% CI: 1.30, 2.78). Women with 3 to 4 children (aOR: 1.26; 95% CI: 0.97, 1.62) and 5 or more children (aOR: 2.42; 95% CI: 1.76, 3.32) were more likely to experience provider encouragement compared to women with 1 to 2 children. Participants who had previously gone in for a 45th-day postpartum health clinic visit had 2.67 times the odds of provider encouragement to use contraception due to providers’ perception of high parity than those who had never gone in for that check-up (95% CI: 2.10, 3.37). Variance inflation factor was less than 3 for all explanatory variables.

**TABLE 3.** Factors Associated With Provider Encouragement to Use Family Planning Due to a Provider’s Perception of Parity in Nouna and Ouagadougou, Burkina Faso

	Adjusted Odds Ratio (95% CI)
Age (ref: 15–24 years), years	
25–34	1.51 (1.11, 2.06)
35–49	1.47 (1.05, 2.07)
Education level (ref: none)	
At least some primary	1.16 (0.90, 1.50)
At least some secondary	1.27 (0.85, 1.90)
Marital status (ref: not married)	
Currently married	1.56 (1.08, 2.26)
Primary mode of transport (ref: foot)	
Bicycle	0.89 (0.68, 1.16)
Motorcycle	0.53 (0.36, 0.80)
Car	0.92 (0.37, 2.25)
Number of children (ref: 1–2)	
3–4	1.26 (0.97, 1.62)
5+	2.42 (1.76, 3.32)
Site (ref: Ouagadougou)	
Nouna	1.90 (1.30, 2.78)
Ever had a 45th day postpartum visit (ref: no)	
Had a 45th day postpartum visit	2.67 (2.10, 3.37)

Abbreviation: CI, confidence interval.

## DISCUSSION

In this article, we build on previous work on FP provider bias to expand its definition. We draw a distinction between downward provider bias, in which providers hinder FP access by withholding and limiting services, and upward provider bias, in which providers pressure or encourage FP clients to adopt a contraceptive method. Using cross-sectional survey data collected among reproductive-aged women in Burkina Faso as a case study, we then quantitatively measured 1 form of each upward and downward provider bias: encouragement or discouragement from a provider to adopt a contraceptive method due to a provider’s perception of high or low parity. Our findings reveal that many women with children in Burkina Faso experienced this form of upward provider bias, with 16% of respondents reporting provider encouragement to use contraception because they were told they had “too many” children. Far fewer reproductive-aged women (1%) reported ever being discouraged from using contraception because they did not have “enough” children. Living in a rural area, being older, having more children, being married, and having attended a 45th-day postpartum health clinic visit were all associated with increased odds of experiencing provider encouragement to use contraception due to provider perception of high parity.

Our findings reveal that 16% of women with children in our sample reported experiencing provider encouragement to use contraception because the provider perceived them to have “too many” children.

Age, marital status, and parity are often discussed as motivators for downward provider bias, with methods or services being withheld from younger, nulliparous, or unmarried women.^4^^,^^7^^,^^12^^,^^28^ Our study finds that these tendencies seem to hold true for upward bias in the inverse, revealing that women who were older, married, and had higher parity had increased odds of experiencing provider encouragement to use contraception due to perceptions of high parity compared to their younger, unmarried, or lower parity counterparts. Thus, we posit it is possible (and even likely) that upward and downward biases coexist in the same programs and settings, with FP services being denied to certain sociodemographic groups and foisted upon others.

These findings coincide with previous studies that show that rural women and multiparous women may be more likely to be targeted for fertility control.^29^^,^^30^ Interventions are often implemented with the goal of increasing access to contraceptive methods or services and may inadvertently promote upward provider bias through a narrow focus on measuring contraceptive uptake.^18^ This may be especially true in contexts that use target-based approaches to fertility control. For example, the Burkinabè government adopted its “National Plan for Accelerating Family Planning” in 2017 with the goal of increasing the modern contraceptive prevalence rate to 32% by 2020.^31^ This plan was in place during data collection for the present study and may contribute, in part, to the prevalence of provider encouragement to use contraception based on provider perceptions of parity. Within this broader context, we postulate that the present findings may be part of a larger pattern of upward provider bias in programs seeking to maximize contraceptive uptake and limit population growth.^17^^,^^32^

We also find that having attended a 45th-day postpartum check-up was associated with experiencing provider encouragement to use contraception, even after controlling for parity. Over 40% of women who attended a 45th-day postpartum visit were asked to choose a contraceptive method without being first asked if they wanted to use contraception at all. These findings may indicate that some women in Burkina Faso experience biased and/or directive counseling during postnatal care. We note that our data are limited in that we are unable to determine whether upward provider bias occurred at the postpartum visit or whether women who attend the 45th-day postpartum visit are more likely to use health services and therefore have more opportunities to experience upward provider bias. Further, we asked participants whether they had ever attended a 45th-day postpartum visit, which may result in recall bias. The postpartum visit is a critical juncture in women’s reproductive lives and, therefore, a prime opportunity to reach women who want to use contraception and may otherwise not receive contraceptive services.^33^ Thus, future research to develop and pilot new interventions that train providers in person-centered postpartum contraceptive counseling is necessary to promote contraceptive autonomy among reproductive-aged women with children.

### Strengths and Limitations

In our case study, measurement of provider encouragement or discouragement to use or not use contraception due to provider perceptions of parity was dependent on women’s self-reported perceptions of their interactions with FP providers. In this measurement approach, rather than eliciting details about specific provider interactions and determining, as third-party researchers, whether these were valid or biased, we trusted participants to share their interpretations of their experiences. These reported perceptions of encouragement and discouragement shed light on how women interact with FP service providers by centering the patient and their thoughts and feelings. Such person-centered measurement approaches complement other data collection techniques that quantify provider bias, such as mystery clients, in-depth interviews, and provider surveys.^34^ Triangulation across these techniques could provide a more complete picture of client-provider interactions. To gain a fuller and more holistic understanding of upward provider bias, further measurement approaches and data collection tools should be employed.

While we believe that our attempt to measure specific forms of upward and downward FP provider biases is a step in the right direction toward improving person-centered measurement in FP research, this measurement approach will benefit from future refinement and development. For example, in this case study, we measure only a certain type of bias: provider encouragement or discouragement to use or not use contraception due to providers’ perception of parity. Our measures are not nearly comprehensive of the range and spectrum of provider biases that patients experience when receiving health services. Further, our measurement approach relies on just 2 questions to capture lifetime experiences of provider encouragement and discouragement to use or not use contraception due to providers’ perception parity. Not only is this approach subject to measurement error due to recall bias, but it also misses nuance and context by simplifying these experiences into just 2 questions with binary answers. Additionally, capturing lifetime prevalence of these experiences may limit the programmatic usefulness of these measures, as it is unclear exactly when and how these experiences occurred. We also note that our findings are specific in place and time; upward and downward provider biases vary by context, and we cannot have a holistic picture of how these forms of bias operate without a broad understanding of their occurrences in a range of settings.

### Future Steps

We view this case study as a call to action for future work to explore the ways that bidirectional forms of FP provider bias operate. One important first step of this work is to improve measurement of upward provider bias using a range of data collection techniques. We encourage the development of new, more comprehensive measures of upward and downward provider biases that: (1) capture different types of these biases, including a range of biases along the spectrum of more subtle to more overt, (2) ask more nuanced questions about where and how bias occurs and how it influences contraceptive decision-making, and (3) relate biases to a specific time and place, such that interventions can be developed to address such biases.

## CONCLUSIONS

This study builds on our understanding of provider bias by describing the distinction between upward and downward FP provider biases and capturing the prevalence of provider encouragement to use or discouragement from using contraception based on perceptions of parity among reproductive-aged women in a population-based survey. Our case study findings reveal that experiencing encouragement to use contraception from providers based on perceptions of high parity is fairly common in our sample and experiencing discouragement from using contraception based on perceptions of low parity is considerably less common. Therefore, we find that downward provider bias—the more conventional understanding of provider bias in which providers hinder access to contraception—was considerably less prevalent in our sample than our novel conception of upward provider bias, in which providers encourage or emphasize contraceptive use. Those with more children, who reside in rural areas, and who attend postpartum health clinic visits may be especially vulnerable to such bias. This type of provider bias is an understudied threat to contraceptive autonomy, preventing women from making free, full, and informed choices about how many children to have and if, when, and how to prevent pregnancy. Fully understanding the mechanisms through which upward provider bias operates is necessary to promote person-centered FP programs and policies.

## Notes

**Peer Reviewed**

**First published online:** June 13, 2023.
